# Stem Cell Therapy in Dengue Virus-Infected BALB/C Mice Improves Hepatic Injury

**DOI:** 10.3389/fcell.2021.637270

**Published:** 2021-07-05

**Authors:** S. Sakinah, Sivan Padma Priya, Pooi Ling Mok, Rusheni Munisvaradass, Seoh Wei Teh, Zhong Sun, Badr Alzahrani, Faizal Abu Bakar, Hui-yee Chee, Rukman Awang Hamat, Guozhong He, Chenglong Xiong, Narcisse Joseph, Jia Bei Tong, Xiaoyun Wu, Mahendran Maniam, Antony V. Samrot, Akon Higuchi, S. Suresh Kumar

**Affiliations:** ^1^Department of Medical Microbiology, Faculty of Medicine and Health Sciences, Universiti Putra Malaysia, Seri Kembangan, Malaysia; ^2^Department of Clinical Laboratory Sciences, College of Applied Medical Sciences, Jouf University, Sakakah, Saudi Arabia; ^3^Department of Biomedical Sciences, Faculty of Medicine and Health Sciences, Universiti Putra Malaysia, Seri Kembangan, Malaysia; ^4^Bioinformatics and Computational Biology, Malaysia Genome Institute, National Institute of Biotechnology Malaysia (NIBM), Kajang, Malaysia; ^5^Institute of Health, Kunming Medical University, Kunming, China; ^6^Department of Medical Microbiology, School of Public Health, Fudan University, Shanghai, China; ^7^First Affiliated Hospital of Baotou Medical College, Inner Mongolia University of Science and Technology, Baotou, China; ^8^Sultan Idris Education University, Tanjong Malim, Malaysia; ^9^School of Bioscience, Faculty of Medicine, Bioscience and Nursing, MAHSA University, Jenjarom, Malaysia; ^10^Department of Chemical and Materials Engineering, National Central University, Taoyuan City, Taiwan; ^11^R&D Center for Membrane Technology, Chung Yuan Christian University, Taoyuan City, Taiwan; ^12^Centre for Materials Engineering and Regenerative Medicine, Bharath Institute of Higher Education and Research, Chennai, India

**Keywords:** dengue infection, stem cell therapy, DENV 2, next-generation sequencing, hepatology

## Abstract

Extensive clinical efforts have been made to control the severity of dengue diseases; however, the dengue morbidity and mortality have not declined. Dengue virus (DENV) can infect and cause systemic damage in many organs, resulting in organ failure. Here, we present a novel report showing a tailored stem-cell-based therapy that can aid in viral clearance and rescue liver cells from further damage during dengue infection. We administered a combination of hematopoietic stem cells and endothelial progenitor cells in a DENV-infected BALB/c mouse model and found that delivery of this cell cocktail had improved their liver functions, confirmed by hematology, histopathology, and next-generation sequencing. These stem and progenitor cells can differentiate into target cells and repair the damaged tissues. In addition, the regime can regulate endothelial proliferation and permeability, modulate inflammatory reactions, enhance extracellular matrix production and angiogenesis, and secrete an array of growth factors to create an enhanced milieu for cell reparation. No previous study has been published on the treatment of dengue infection using stem cells combination. In conclusion, dengue-induced liver damage was rescued by administration of stem cell therapy, with less apoptosis and improved repair and regeneration in the dengue mouse model.

## Introduction

Dengue virus (DENV) infections transmitted by mosquitoes is setting half of the world’s population at risk. Despite advancement in biomedical sciences in the past decades, dengue morbidity and mortality have not declined. Each year, 390 million people are infected with dengue fever, and 96 million of them have different degrees of clinical manifestations (Bhatt et al., 2013). There are currently no specific treatments and substantial vector control measures to prevent their rapid emergence and global spread. As a result, the global incidence of dengue fever has increased dramatically in recent decades, and it is urgent to introduce a new alternative therapy to overcome dengue. Here, we show a novel therapeutic approach using a combination of stem cells [hematopoietic stem cells (HSCs) and endothelial progenitor cells (EPCs)] to treat DENV infection and rescue clinical outcome and dengue-induced liver injury in an animal model. The focal points studied were changes in the state of the animals, clinical profiles, histopathology, and next-generation sequencing (NGS) of each liver gene expressed in the control, DENV-infected (DVI), and stem-cells-treated (DVI-SCT) groups. To that effect, the therapeutic efficacy of stem cells against viral infection was evaluated by observing and examining several physical parameters, including behavioral changes exhibited by the experimental animals, clinical profile parameters (i.e., platelet, white blood cell, red blood cell counts, and lymphocyte, and hemoglobin levels), histopathological changes, and NGS data. The findings demonstrate that the use of combination stem cell therapy cannot only heal vascular injury, thrombocytopenia, and hepatocyte damage caused by DENV but also reduce the presence of DENV in the liver tissue, suggesting clearance of DENV.

## Results and Discussion

### Behavioral Changes in Experimental Animals

**FIGURE 1:**
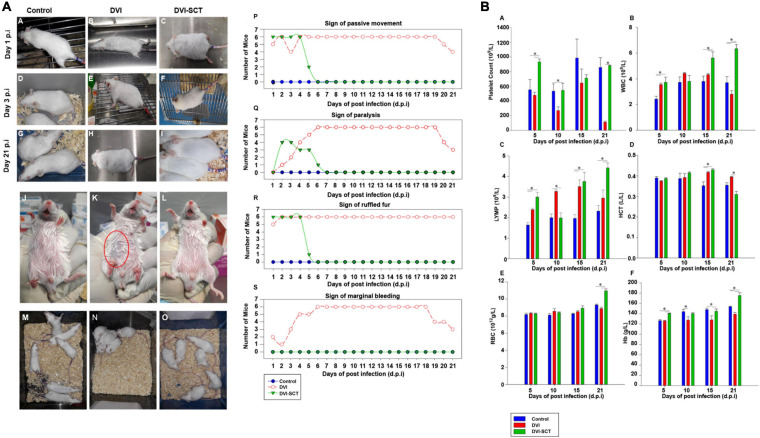
Behavioral changes in experimental animals and clinical profile analysis. **(A)** Mouse behavioral and physical changes. **(a–i)** Mouse fur condition at different times during the experiment. **(j–l)** Sign of hemorrhage. **(m–o)** Mouse mobility. **(p–s)** The number of mice showing physical changes at different dpi. **(p)** Sign of passive movement, **(q)** Sign of paralysis, **(r)** Sign of ruffled fur, and **(s)** Sign of marginal bleeding. (•) Control, (◦) DVI, and (▼) DVI-SCT. **(B)** Comparison of hematological parameters for each group at different days post-infection (dpi). **(a)** Platelet count, **(b)** White blood cells (WBC), **(c)** Lymphocytes (LYMP), **(d)** Hematocrit (HCT), **(e)** Red blood cells (RBC), and **(f)** Hemoglobin (Hb). Legend: Control (blue bar), DVI (red bar), and DVI-SCT (green bar). The data were analyzed using one-way ANOVA, and differences were considered significant when *p* < 0.05 (*).

While other mouse models have been genetically modified and/or immunocompromised, we choose BALB/c mouse model, as it would mimic the normal human condition during the occurrence of dengue infection (Barth et al., 2006). The changes in mice observed throughout the experiment are shown in Figure 1. No death was observed throughout the experiment. The weight of each mouse was plotted in Supplementary Figure 4. There was no significant difference in weight of mice in each group. The mice in the control group were active and showed no signs of hemorrhage and bleeding (Figures 1Aj,m), and the condition of the fur was well-groomed throughout the experiment (Figures 1Aa,d,g). Meanwhile, the mice in the DVI group displayed ruffled fur after dengue infection (Figures 1Ae,h). A marginal purple-bluish spot was found under the abdominal skin area and deemed a sign of bleeding in the DVI group mice (Figure 1Ak). They stayed close to each other and had poor mobility and temporary paralysis of the hind limbs (Figure 1An). The above condition continued until the end of the experiment and did not improve significantly.

On the other hand, the mice in the DVI-SCT group developed the same conditions as the DVI group at the early stage of dengue infection, such as inactivity, poor mobility, and temporary paralysis of the hind limbs (Figure 1Ao) but no signs of bleeding (Figure 1Al). The above symptoms recovered to the state similar to the control group mice, within 1 week after stem cell treatment. At the same time, ruffled fur at first day postinfection (dpi) of dengue infection (Figure 1Ac) was observed to be improved 1 day after stem cell treatment (Figure 1Af), then were well-groomed by the sixth dpi, and remained groomed until the 21st dpi (Figure 1Ai).

Dengue fever can cause muscle pain and muscle weakness, and the mice in the DVI group showed the corresponding poor mobility, hind limb paralysis, and restricted movement. Dengue-induced thrombocytopenia and hemorrhage were also presented as mild bleeding in DVI mice. Furthermore, dengue fever can cause hypovolemic shock, and the phenomenon of ruffled fur due to piloerection, hypothermia, and close proximity to each other in the DVI group is evidenced. After stem cell treatment (SCT), the ruffled fur, paralysis, and mobility all recovered within a few days, and no signs of hemorrhage were observed (Figure 1A). This was likely due to the stem cell treatment, especially with EPCs, which promote endothelial repair and neovascularization, in turn assisting in recovery from paralysis and hemorrhage (McAllister et al., 2013). DENV 2 was inoculated intraperitoneally because we would like to mimic the human dengue-infected signs and also to establish severe dengue infection. Intraperitoneal route establishes signs of dengue diseases even with lower viral dose inoculation including thrombocytopenia, liver damage, hemorrhage, and viremia and disseminated to various organs. In addition, DENV infection of animals by intraperitoneal route also reproduce some aspect of human disease (Paes et al., 2005; Kuruvilla et al., 2007; Zellweger et al., 2017). Moreover, in wild-type mouse models, intraperitoneal DENV-infection results in neurological abnormalities, which is considered as criteria for severe dengue by the World Health Organization (World Health Organization (WHO), 2009). The stem cell treatment was performed via intravenous administration to increase hematopoietic reconstitution in the mice bone marrow. Apart from being the most commonly used technique for intravenous route treatment, it was reported to be safe and had no tumor formations and no cases of infections or increased pain in human trials. In addition, it also helps in rapid diffusion of donor cell population, faster repair of blood vessels, and increase in long-term engraftment without increasing fetal mortality (Geffner et al., 2008; Yang et al., 2015; Boelig et al., 2016).

### Clinical Profile Analysis

Platelet dysfunction is one of the hallmarks of dengue infection, along with pronounced thrombocytopenia. To assess the effects of stem cells treatment on platelets and other blood cells, blood analysis was performed on every group of mice at different dpi (Figure 1B). In this study, the platelet counts in the DVI group were lower than that in the control and DVI-SCT groups. The slight increase on the 15th dpi in the DVI group is suggestive of the body innate immune system trying to recover the platelet counts to a normal level; however, it failed, and the count dropped to a critical level (near zero) by 21st dpi comparable with the platelet count trend by Frias-Staheli et al. (Jiang et al., 2013). Mice in the DVI-SCT group showed an increase in platelet count from fifth dpi and achieved similar level with that of the control group on 21st dpi (Figure 1Ba). It indicated that the administration of the stem cells cocktails during DENV2 infection has restored platelet count to the normal level. The HSCs in the stem cells cocktail can differentiate into megakaryocytes and stimulate bone marrow to produce platelets (Woolthuis and Park, 2016). The administration of growth factor thrombopoietin (TPO) in the treatment also improved platelet production in the DVI-SCT group. In addition, including EPCs in the treatment regimen allows reconstitution of the bone marrow (BM) niche and recovery of hematopoiesis, thus increasing the platelet count (Salter et al., 2009). In addition, the white blood cell (WBC) counts observed in the DVI group in this study showed a slight increase in the initial phase but began to decline after the 15th dpi, which may be related to the BM inhibition effect of DENV similar to the changes reported in a previous dengue infection study (Tan et al., 2011; Pascutti et al., 2016) and the leukopenia state reached at the end of the experiment. In contrast, the WBC count in the DVI-SCT group remained at the same level as the control group at the initial stage and increased significantly on the 15th and 21st dpi compared with those in the DVI group (Figure 1Bb). This indicated that the treatment with HSCs effectively increased and sustained blood cell production, including proliferation and differentiation into WBC.

Similar to changes in WBC counts, lymphocyte counts in the DVI group also increased up to 15th dpi, then began to decrease. However, in the DVI-SCT group, a high lymphocyte count was found as early as the fifth dpi, promoting a faster immune response compared to the DVI group. The lymphocyte count in the DVI-SCT group reached normal levels by the 10th dpi and continued to rise (Figure 1Bc). These results showed that the stem cell therapy can facilitate increased DENV clearance.

No significant difference in the hematocrit (HCT) was observed in the DVI and DVI-SCT groups, except for on the 21st dpi, at which time the DVI group exhibited the highest HCT among all groups. This might be due to dengue-induced vascular leakage, which caused large amount of plasma exudate and increased HCT (Figures 1B,d). High HCT was shown to result in increased blood viscosity and even arterial thrombosis (Salazar Vázquez et al., 2008). Thus, the decreased HCT in the DVI-SCT group by 21st dpi indicated that the stem cell therapy in this study is safe and effective. The platelet, WBC, and HCT data obtained from this study are comparable with several previous studies despite using different treatment materials, DENV strain, titer, and environment (Sreekanth et al., 2014, 2016).

The hemoglobin level and red blood cell (RBC) count in the DVI group were found to be low, whereas their levels in the DVI-SCT group consistently increased from the fifth dpi onward. The RBC level is similar between the groups. However, the RBC and Hb levels in the DVI-SCT group was noticeably increased from the 15th dpi (Figures 1Be,f).

The changes in all blood cells found in this study suggests that HSCs treatment repopulate and regenerate production of blood cells. HSCs CD34^+^ cells are progenitors that have the potential for cell renewal and myeloid and lymphoid differentiation, with their ability to home inflammation and tissue injury sites and aid in its repair (Nagy et al., 2005). In a cell-tracking study, Brudecki et al. (2012) showed that HSPCs were detected in bone marrow after 2 days of transplantation and homed to the peritoneum at a later time around the fifth day. In another study, the donor-derived granulocytes and monocytes were detected after 6 days of HSPCs transplantation into lethally irradiated mice (Akashi et al., 2000). In this study, increased blood cells were detected approximately 8 days after stem cells treatment, suggesting that time is likely required for the transferred HSCs to differentiate into mature immune and other blood cells and to also to mount the protective response in the mice.

### Liver Biochemical Analysis

**FIGURE 2:**
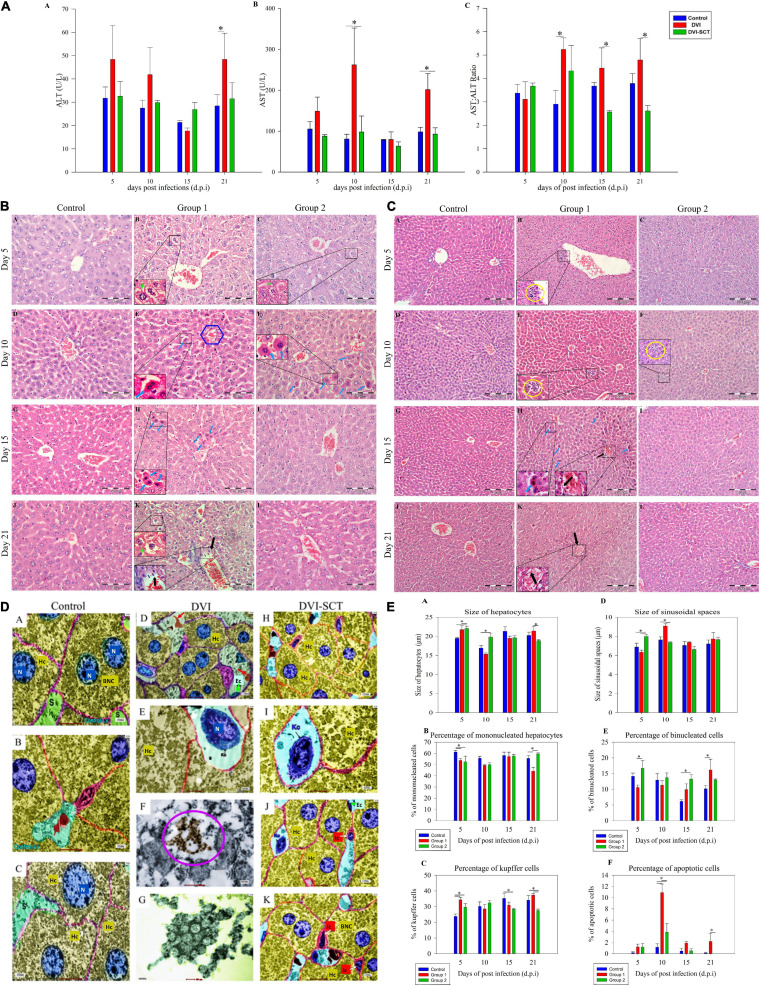
Hepatological analysis. **(A)** Liver biochemical analysis (ALT, AST) at different dpi in different groups. Legend: Control (blue bar), DVI (red bar), DVI-SCT (green bar). **(B)** Comparison of histological examination of liver at different dpi in different groups (× 400 magnification). Legend: Steatosis cells (green star), inflammatory cell infiltration (yellow round), apoptotic cells (blue arrow) and blood vessel tear (black arrow), and central vein (blue hexagon). **(C)** Liver microscopic images at different dpi in different groups (× 200 magnification). Legend: apoptotic cells (blue arrow) and blood vessel tear (black arrow). **(D)** Liver transmission electron microscopy images of DENV2-infected mice. N, nucleus; BNC, binucleated cells; MNC, mononucleated cells; Ec, endothelial cells; Hc, hepatocytes; Kc, Kupffer cells; S, sinusoids; Ic, Ito cell; red arrow, red blood cells; purple circle, DENV particles. **(E)** Cell population counts at different dpi. Legend: Control (blue bar), DVI (red bar), and DVI-SCT (green bar). The data were analyzed using one-way ANOVA, and differences were considered significant when *p* < 0.05 (*).

Aspartate aminotransaminase (AST) and alanine aminotransaminase (ALT) are crucial markers in detecting liver damage, with ALT being the most prominent indicator. In this study, the AST level was significantly increased in the DVI group on the 10th and 21st dpi. In contrast, it is worth noting that in the DVI-SCT group, the AST level was maintained near to that in the control group throughout the experiment (Figure 2Ab). Meanwhile, the ALT level was highest in the DVI group except on the 15th dpi, with an increase from 30 to 50 compared to that in the control group on the 21st dpi. A pilot study by Kuo et al. (1992) showed that AST and ALT level were altered at 82.2 and 93.3% in a total case of 270 dengue fever, while another study revealed 50 cases with higher ALT level in DHF patient. However, the symptomatic dengue-infected patients showing hepatic enzyme abnormalities are able to recover faster (Seneviratne et al., 2006). Animal model studies found that the peak for both AST and ALT levels is identified, which is similar to human cases and decreased after 2 weeks (Kuo et al., 1992; Franca et al., 2010). Similar to other human and animal study, we also observed increased and decreased ALT level at 15th dpi. However, failure in recovery suggest the rise in ALT and AST levels again on the 21st dpi. Conversely, in the DVI-SCT group, the ALT level was successfully maintained with the control (Figure 2Aa). In our findings, an AST/ALT ratio indicating liver injury was obviously detected in the DVI group from 10th dpi onward. A slight increase in liver injury was observed in the DVI-SCT group on the 10th dpi, but the liver condition was maintained at par with that in the control from the 15th dpi onward (Figure 2Ac), indicating that DENV-induced liver damage has recovered after stem cell therapy.

### Microscopic Analysis of Liver Tissue

Liver manifestations are not just related to viral toxicity but are also due to the immune reaction against the virus, often damaging an individual’s own tissues in response to intracellular viral particles (Samanta and Sharma, 2015). Livers of control mice injected with Eagle’s minimum essential medium (EMEM) and phosphate buffered saline (PBS) did not display any apoptosis and structural alteration (Figure 2Ba,d,g,j) on the 5th (Figure 2Ca), 10th (Figure 2Cd), 15th (Figure 2Cg), or 21st dpi (Figure 2Cj).

In the DVI group, the hepatic injury was observed in all the DVI mice, and the histopathological examination showed generalized steatosis (i.e., lipid accumulation within hepatocytes), tumefaction, mild apoptosis, and inflammatory cell infiltration on the fifth dpi (Figure 2Cb). These injuries were initially mild but become severe at the 10th dpi (Figures 2Be,2Ce). At 10th dpi, the apoptosis was also increasing, characterized by a round or oval mass having an intense eosinophilic cytoplasm and dense nuclear chromatin fragments. On the 15th dpi, the number of apoptotic hepatocytes was reduced, but the signs of steatosis were predominant, with increases in hepatocyte diameters (Figures 2Ch,2Bh). Furthermore, hepatic injury was more obvious on the 21st dpi (Figure 2Ck), with significantly higher hepatocyte diameters than those observed in the control and DVI-SCT groups. Other changes include increased hepatocyte swelling, blood vessel dissection, inflammatory cell infiltration near the blood vessels, diffuse steatosis, and mild hemorrhage (Figure 2Ck). The findings can be correlated with cytokine-mediated defense against the viral-infected or target cells. These immune reactions are primarily mediated by T cells and related interacting cells that secrete antibodies against ECs, finally resulting in the cytokine “Tsunami” acting on dengue target cells (Samanta and Sharma, 2015). These findings were comparable to human DV cases (Lawn et al., 2003; Basilio-de-Oliveira et al., 2005) and several animal models experiments (Barth et al., 2006).

In the DVI-SCT group, the liver cells also displayed diffuse steatosis, hepatocyte swelling, tumefaction, increased hepatocyte diameter, inflammatory cell infiltration, and rare blood vessel dissection with no hemorrhagic signs (Figure 2Cc) on the 5th dpi. On the 10th dpi, less inflammatory cell infiltration and no hepatocyte swelling were recorded (Figure 2Cf). Additionally, recovered tumefaction, mild steatosis, and a reduction in apoptotic cell number were seen in the DVI-SCT group (Figure 2Cf) compared with the DVI group. All signs were reduced in the DVI-SCT group by the 15th dpi, and subsequently, no inflammatory cell infiltration, hepatocyte swelling, or bleeding was observed, along with mild steatosis (Figure 2Ci). The binucleated cell percentage was consistently maintained at a value similar to that in the control until the 21st dpi (Figure 2Cl). In addition, recovered tumefaction, mild steatosis, and a reduction in apoptotic cell number were on the 10th dpi (Figure 2Bf). From the 15th dpi, no inflammatory cell infiltration, hepatocyte swelling, or bleeding was observed, along with less steatosis (Figure 2Bi). The binucleated cell percentage was consistently maintained at a value similar to that in the control until the 21st dpi (Figure 2Bl).

The stem cells injection induces liver tissue regeneration and repopulation by paracrine action of the injected cells (Lozito et al., 2009). Several growth factors and cytokines released during liver injury stimulate the migration of bone marrow cells (HSCs) to the injury site through circulation and hepatogenic differentiation and populate the liver after intravenous transplantation (Lagasse et al., 2000; Zhang et al., 2003; Ruhnke et al., 2005). Apart from that, EPCs from the bone marrow also reside at the sinusoidal endothelium (Kallis et al., 2007). Several animal studies have demonstrated that EPCs from the bone marrow have been incorporated into the sinusoidal endothelium and further applied for the reconstruction of hepatic sinusoids during liver regeneration with the elevated level of hepatocyte growth factor (HGF) and vascular endothelial growth factor (VEGF) (Fujii et al., 2002; Taniguchi et al., 2006) and hence overall repaired the injured liver after dengue infection.

### Transmission Electron Microscopy of Liver Tissue

The transmission electron microscopy (TEM) observations of the liver tissue detailed the presence of mononucleated and binucleated cells and the variations in the size of the nucleus in the hepatocytes (Figure 2D). Compared with the control group, the DVI group showed complex viral-induced subcellular changes. The hepatocytes exhibited mononucleated cells (MNC), binucleated (BNC), Kupffer cells, and Ito cells, indicating both inflammatory response and the reparative responses (Figures 2Dd,e). Furthermore, the sinusoidal spaces were more frequently noted to contain Kupffer cells in the DVI group than in the DVI-SCT group. This also demonstrates the increased immune reaction in the DVI-SCT group due to the dominating actions of the virus. The DVI-SCT group exhibited a relatively larger cell size at the same magnification than the control and DVI groups, which indicates tumefaction (Figures 2Dh,j,k). Virus-like particles were observed inside hepatocytes in the DVI group (Figures 2Df,g) but not in the hepatocytes in DVI-SCT group after careful evaluation of the sectioned area. These results provide evidence of the relative clearance of the virus in the DVI-SCT group.

### Liver Cell Population Count

Livers of control mice showed a significant increase in hepatocyte diameter alongside diminished sinusoidal space (Figures 2Ea,b). Predominantly mononucleated hepatocytes with less binucleated hepatocytes and an increased number of Kupffer cells were also demonstrated in comparison with the control group (Figures 2Ed,e,f) (*p* > 0.05). On the 10th dpi, reduced hepatocyte diameter and extended sinusoidal space were observed along with the presence of diffuse hepatocyte apoptosis near the central vein (Figures 2Eb,c).

On the 21st dpi, the percentage of binucleated hepatocytes and Kupffer cells was significantly higher in the DVI group than in the control and DVI-SCT groups (Figures 2Ee,f). In the DVI-SCT group, the percentage of binucleated cells was at its highest on the fifth dpi (Figure 2Ee), alongside wider sinusoidal spaces (Figure 2Eb) and a lower percentage of Kupffer cells (Figure 2Ef), compared with the DVI group. In the DVI-SCT group at the 15th dpi, quantification of the apoptotic cells yielded a lower percentage compared with that in the DVI group, and the number consistently remained near that of the control level until the 21st dpi (Figure 2Ec). Furthermore, the percentage of Kupffer cells was significantly lower than that in the control group and only slightly lower than that in the DVI group (Figure 2Ef). In addition, the size of the hepatocytes and the percentage of mononucleated hepatocytes in the DVI-SCT group was not significantly different than those in the control and DVI groups on the 15th dpi. Nevertheless, the DVI-SCT group exhibited reduced liver injury by the 21st dpi, as well as reduced hepatocyte size (characterized by swelling and rounding up of the hepatocyte which leads loss of hepatocyte shape), a significantly higher mononucleated hepatocyte percentage, and a lower percentage of Kupffer cells (Figures 2Ea,d,f).

The low number of binucleated hepatocytes in the DVI group at early stage indicated that liver recovery phase occurs only after 21st dpi. This may be one of the reasons for the mortality of dengue due to liver failure. In contrast, the number of binuclear cells in the DVI-SCT group achieved its peak on the fifth dpi and thereafter remained at a level higher than in the control group, suggesting that the liver repair has begun in the early stages of infection (Woolthuis and Park, 2016). In addition, the important role of Kupffer cells in infection and inflammation explains their high levels in the DVI group, suggesting that viral activity persisted until the end of the experiment (Paes et al., 2009). In contrast, the DVI-SCT group showed a lower percentage of Kupffer cells throughout the experiment. The results supported that the combined stem cell therapy contributes to liver regeneration and recovery during DENV infection.

### Immunohistochemical Study of Liver Tissue

**FIGURE 3:**
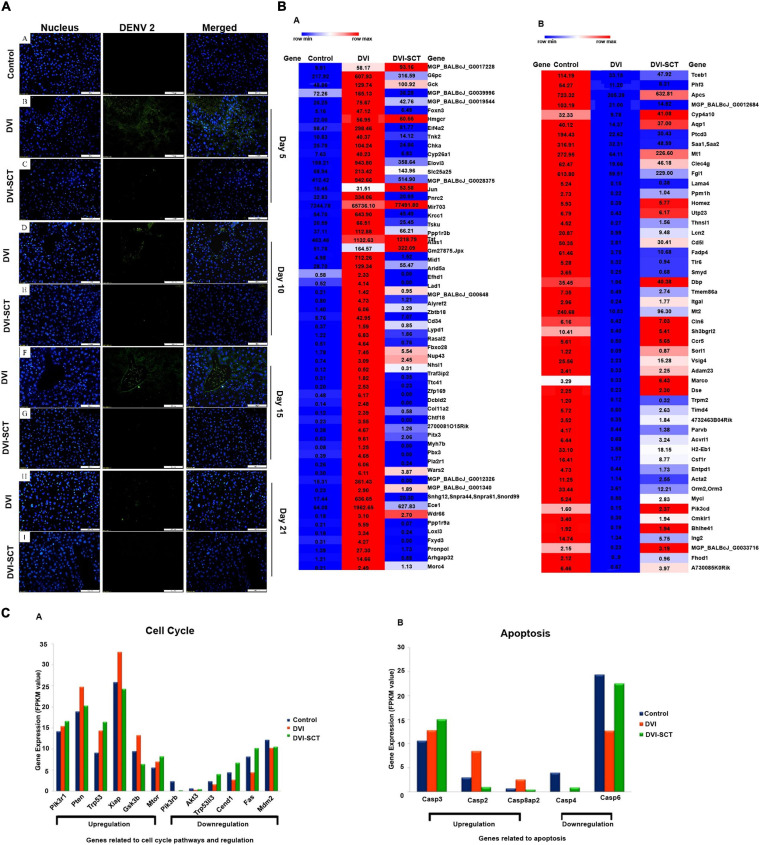
Immunohistochemical assessment and next-generation sequencing. **(A)** Immunohistochemical study of DENV-infected BALB/c mice liver at different dpi. Liver sections were captured at a magnification of × 200 under a fluorescence microscope. **(a)** Control group, **(b,c)** liver sections of DENV-infected and stem-cells-treated mice on the 5th dpi, **(d,e)** 10th dpi, **(f,g)** 15th dpi, and **(h,i)** 21st dpi. Blue (DAPI-stained nuclei) and green (Alexa Fluor stained DENV antigen). The green fluorescence seen in the DVI group was diminished in the DVI-SCT group. **(B)** Heatmap of upregulated genes. **(C)** Heatmap of downregulated genes. The dysregulation (up - or downregulation) of liver genes was quantified in three sets of BALB/c mice: control mice injected with Eagle’s minimum essential medium (EMEM) and phosphate-buffered saline (PBS); DVI mice infected with DENV2; and DVI-SCT mice infected with DENV2 and treated with HSCs, EPCs, and growth factor (*n* = 3). The heat maps generated were quantified based on the row minimum and row maximum values (specific for each gene), with red indicating genes highly upregulated and blue depicting genes downregulated. **(a)** Gene expression related to cell cycle pathways and regulation. The genes *Pik3r1*, *Pten*, *Trp53*, *Xiap*, *Gsk3b*, and *Mtor* showed gene expression upregulation following dengue virus infection (DVI), which were either corrected to reflect the control levels (*Pten*, *Xiap*, and *Gsk3b*) or continuously upregulated to benefit mice (*Pik3r1*, *Trp53*, and *Mtor*) upon stem cell treatment (DVI-SCT). Meanwhile, the genes *Pik3r6*, *Akt3*, *Trp53i13*, *Ccnd1*, *Fas*, and *Mdm2* showed downregulation upon dengue virus infection (DVI), which was also all corrected to reflect control values upon stem cell treatment (DVI-SCT). **(b)** Upregulated and downregulated genes related to apoptosis. The genes *Casp3*, *Casp2*, and *Casp8ap2* showed upregulated gene expression following dengue virus infection (DVI) and was corrected to reflect control levels (*Casp2* and *Casp8ap2*) or continuously upregulated to benefit mice (*Casp3*) upon stem cell treatment (DVI-SCT). Meanwhile, the genes *Casp4* and *Casp6* showed downregulation upon dengue virus infection (DVI), which was corrected to reflect control levels upon stem cell treatment (DVI-SCT).

To assess the ability of the stem cell cocktail infusion in eliminating DENV from dengue-infected mice, immunohistochemical staining was performed to detect the presence of viral antigen in livers of DVI and DVI-SCT mice. Immunohistochemistry showed that mouse hepatocytes positive for DENV antigens (green fluorescence) were more prominent in the DVI group than in the DVI-SCT group throughout the experiment (Figure 3A). Despite the slight reduction in DENV antigens observed in the DVI group by the 21st dpi, the virus was not completely cleared from the liver cell population. In contrast, the DVI-SCT group displayed minimal expression of DENV antigens, suggesting that the virus was eliminated from the liver cells by the 21st dpi. Here, we have provided a qualitative data on the presence of virus in infected mice. It is also very important to provide quantitative data to have a stronger evidence of viral clearance. Thus, dengue virus screening assays, such as immunofluorescence assay, IC50, FFU assay, quantitative reverse transcription PCR (RT-PCR) assay, and plaque assay method, are ample and fit for the high-throughput screening of virus clearance (Low et al., 2011; Sreekanth et al., 2019).

HSCs are urged by the signals of innate and adaptive immunity in order to respond to the pathogen-specific infection through systematic cytokine stimulation. HSCs express various signaling receptors, which instantly engaged in the infection responses, by its attachment with the infectious ligands and cytokines such as toll-like receptors (TLRs), tumor necrosis factor alpha (TNF) receptor, and interferon (IFN) receptors (Baldridge et al., 2010). The infection signal produced by DENV activates the HSCs and surge the differentiation of HSCs. This differentiation generates the immune effector cells and eventually neutralizes the early infections (Baldridge et al., 2011). HSC also modifies the cytokine secretion profile of T-naive cells, NK cells, effectors, and dendritic cells to induce a tolerant phenotype, secretion of more proinflammatory and anti-inflammatory cytokines, TNF-α, and decreased IFN-γ, with suppressively stimulated IL-4 and IL-10 (Lozito et al., 2008). Some evidence suggests that HSCs act as anti-inflammatory and immunoregulatory (Lozito et al., 2008). A recent study demonstrated treatment for COVID-19 infection using HSCs. The study suggested that the mechanism of HSC to eliminate viruses is by reducing inflammatory cytokines and by increasing anti-inflammatory cytokines that make the virus inactive (Frias-Staheli et al., 2014; Purwati Sumorejo et al., 2020).

Moreover, HSCs also reconstitute and differentiate into immune cell populations in the liver (Jiang et al., 2013), which initiate the phagocytosis of virus and its clearance from the cell. Previous reports have demonstrated the direct dialogue between HSCs outside niches and the immune system through “in” signaling and “out” signaling. The DENV as an “in” signal stimulates the differentiation of HSCs into immune cells (Zheng et al., 2011), which may explain the mechanism of viral clearance seen in the DVI-SCT group in this study. Our findings further validated the ability of the transplanted stem cells to eliminate DENV from the host cells. Although the virus is not visible after stem cells treatment, the mechanism at which stage of the viral replication does the treatment combats is yet to be clarified.

### Gene Expression Profiling Using MiSeq Next-Generation Sequencing

To further analyze the molecular level changes induced by DENV infection and stem cell treatment in dengue-infected mice, RNA sequencing (RNA-seq) using MiSeq next-generation sequencing was employed in this study. Transcriptomics analysis comparing gene expression patterns in different experimental groups showed that of the 38,167 genes expressed in the control group (total fragments per kilobase of exon per million mapped reads, FPKM = 0) and the DVI group (FPKM > 10), 15 were novel genes expressed exclusively in DVI samples, thus suggesting that these 15 genes are triggered by dengue infection (Table 1). These novel genes maybe one of the reasons for the cell dysfunction during dengue infection. Similarly, the expression of novel genes between the DVI and DVI-SCT groups were cross-checked to elucidate the presence of novel genes triggered exclusively by the stem cell treatment. Of the total 38,166 genes expressed, 38 newly expressed genes were identified between the control (FPKM = 0) and DVI-SCT (FPKM > 10) groups (Table 2). These novel genes triggered by the stem cell treatment may be responsible for DENV clearance and for reversing the effects of DENV infection. The occurrence of novel gene expression triggered by either DENV2 infection or stem cell treatments can serve as a vital foundation for future studies. Research on these newly expressed genes may aid in elucidating the interactions between DENV and host or the role of stem cells in DENV clearance.

**TABLE 1:** Novel gene expression in DVI group upon dengue infection compared to control group (novel gene expression triggered by DENV).

Test ID	Gene ID	Gene	Locus	Control (FPKM value)	G1 (FPKM Value)	Log_2_(fold change)	*q* value	Significant
XLOC_013318	XLOC_013318	Rab26os	17:23031320-23077642	0	10.1387	inf	1	no
XLOC_015110	XLOC_015110	Gm10269	18:17819189-17819561	0	16.6601	inf	1	no
XLOC_015371	XLOC_015371	Gm23119	18:72334875-723350 15	0	150.436	inf	1	no
XLOC_016654	XLOC_016654	MGP_BALBcJ_G0025497	2:25678689-25679096	0	10.4326	inf	1	no
XLOC_0l6928	XLOC_0l6928	Gca, MGP_BALBcJ_G0012409, MGP_BALBcJ_G00	2:600892 16-60638515	0	11.9119	inf	1	no
XLOC_021941	XLOC_021941	12410	3:133780090-134207362	0	11.5784	inf	1	no
XLOC_024848	XLOC_024848	4930431Fl2Rik	5:42612105-42624487	0	12.5258	inf	1	no
XLOC_025181	XLOC_025181	Mthfd21	5:89281051-89367605	0	13.6307	inf	1	no
XLOC_029193	XLOC_029193	Igkv8-24	6:67945643-68111408	0	16.0968	inf	1	no
XLOC_030771	XLOC_030771	MGP_BALBcJ_00032442	7:83517729-83518781	0	10.1687	inf	1	no
XLOC_031177	XLOC_031177	Mylpf	7:127702053-127707439	0	120.826	inf	1	no
XLOC_031699	XLOC_031699	Zfp940	7:28205575-2823197 1	0	27.239	inf	1	no
XLOC_033124	XLOC_033124	MGP_BALBcJ_00004533	8:66603202-66686224	0	18.0628	inf	1	no
XLOC_035606	XLOC_035606	Gm44335	9:41376750-41378134	0	574.563	inf	1	no
XLOC_036555	XLOC_036555	MGP_BALBcJ_00014446	X:6 l 059408-61059666	0	23.7736	inf	1	no

**TABLE 2:** Novel gene expression in DVI-SCT group compared to DVI group (novel gene expression triggered by stem cell treatment).

Test ID	Gene ID	Gene	Locus	Control (FPKM value)	G2 (FPKM value)	Log_2_(fold change)	*q* value	Significant
XLOC_003186	XLOC_003186	Sult3a 1	10:31283423-3 1298517	0	48.8338	inf	0.007606	yes
XLOC_021585	XLOC_021585	Hao2	3:96548755-96566667	0	36.539	inf	0.007606	yes
XLOC_021924	XLOC_021924	MGP_BALBcJ_00006282	3:131637308-13 1643455	0	41.0333	inf	0.007606	yes
XLOC_30257	XLOC_30257	MGP_BALBcJ_G0040533	7:24101650-24108542	0	52.1268	inf	0.007606	yes
XLOC_30258	XLOC_30258	Cyp2b9	7:24185787-24223302	0	167.403	inf	0.007606	yes
XLOC_030259	XLOC_030259	MGP_BALB cJ_G0031867	7:24292602-2433421 1	0	209.279	inf	0.007606	yes
XLOC_031499	XLOC_031499	Sult2a3	7:11494777 -11551540	0	34.5995	inf	0.007606	Yes
XLOC_000447	XLOC_000447	Gm27512	1:61169239-61202084	0	436.136	inf	1	no
XLOC_000503	XLOC_000503	Gm25360	1:70252534-70252725	0	94.1518	inf	1	no
XLOC_001981	XLOC_001981	MGP_BALBcJ_G0010132	1:58438442-58523586	0	33.326 1	inf	1	no
XLOC_002729	XLOC_002729	Fmo3	l :161974097-162100359	0	110.528	inf	0.338451	no
XLOC_003112	XLOC_003112	MGP_BALBcJ_G0036352	10:15144200-15155939	0	29.8837	inf	1	no
XLOC_003209	XLOC_003209	Rnu3a	l 0:37833676-37833890	0	116.346	inf	1	no
XLOC_005084	XLOC_005084	Gml7305	11:67460712-67487429	0	10.2884	inf	1	no
XLOC_005422	XLOC_005422	MGP_BALBcJ_G0018893	11:92952527-92956552	0	10.5147	inf	1	no
XLOC_007533	XLOC_007533	Acot3	12:81841321-81863070	0	30.5984	inf	0.478207	no
XLOC_007547	XLOC_007547	Gm 17139	12:82747202-82820080	0	14.7918	inf	1	no
XLOC_012625	XLOC_012625	Gml 0076	16:468038-468405	0	14.5565	inf	1	no
XLOC_013281	XLOC_013281	Gml0226	17:19869811-19870066	0	10.0665	inf	1	no
XLOC_014038	XLOC_014038	Snora78	17:23277562-2327 7996	0	377.441	inf	1	no
XLOC_014192	XLOC_014192	MGP_BALBcJ_G0003360	17:32930035-353 87947	0	17.1602	inf	l	no
XLOC_016013	XLOC_016013	Slc22a26	19:4653942-4675569	0	19.7565	inf	l	no
XLOC_016549	XLOC_016549	mt-Tsl	2:19329205-19618562	0	288021	inf	l	no
XLOC_017204	XLOC_017204	MGP_BALBcJ_G00364 14	2:88663442-88687150	0	11.3819	inf	l	no
XLOC 017433	XLOC 017433	MGP_BALBcJ_G0011813	2:119439014-119486495	0	10.9909	inf	1	no
XLOC_018260	XLOC_018260	MGP_BALBcJ_G0036413	2:26820995-26941552	0	19.9283	inf	l	no
XLOC_020938	XLOC_020938	Gm33051	3:25629719-25640724	0	37.8406	inf	1	no
XLOC_022066	XLOC_022066	Gm24494	3:151934096-151939819	0	52395.6	inf	1	no
XLOC_023192	XLOC_023192	Gm l3203	4: 146653969-146656880	0	10.6962	inf	1	no
XLOC_024169	XLOC_024169	Gm26716	4: 127942608-127976912	0	12.0673	inf	1	no
XLOC_029178	XLOC_029178	Igkv5-39	6:67607309-67607863	0	11.8116	inf	1	no
XLOC_029675	XLOC_029675	Gm44096	6:122520707-122613222	0	17.595	inf	l	no
XLOC_030266	XLOC_030266	Cyp2gl	7:24971909-24994733	0	18.6062	inf	1	no
XLOC_031497	XLOC_031497	MGP_BALBcJ_G003165S,Sult2a2	7:11303713-11394048	0	177.379	inf	0.338451	no
XLOC_031503	XLOC_031503	Sult2a6	7: 11656322-11692614	0	153.433	inf	0.338451	no
XLOC_033861	XLOC_033861	Gm28063	8:54953411-54956948	0	14.4561	inf	1	no
XLOC_034394	XLOC_034394	Gm24357	9: 12002860-12002934	0	145893	inf	1	no
XLOC_035890	XLOC_035890	Rn7sk	9:75583363-75583694	0	11.4497	inf	1	no

Thereafter, the changes in gene expression profiles between groups were calculated by the Cuffdiff method. The genetic profiles demonstrated both upregulation and downregulation of certain liver-associated genes in mice of all three groups. After analysis, it was found that 59 genes were significantly upregulated in the DVI group, and in the DVI-SCT group, 47 of the genes were successfully corrected similar to that of control group, while the remaining genes showed downregulation (Figure 3Ba and Supplementary Table 2). Figure 3Ba shows 59 significantly upregulated genes following DENV infection, among which 47 genes (e.g., *G6pc*, *MGP_BALBcJ_G0039996*, *MGP_BALBcJ_G0019544*, *Foxn3*, and *Eif4a2*) were successfully corrected to preinfection (control) levels, while the rest (e.g., *MGP_BALBcJ_G0017228*, *Gck*, *Hmgcr*, and *Jun*) showed insignificant levels of upregulation. On the other hand, 51 genes were significantly downregulated in the DVI group, and 30 of the genes were successfully corrected to the control level in the DVI-SCT group, while the remaining genes showed a downregulation level (Figure 3Bb and Supplementary Table 1). Figure 3Bb shows 51 significantly downregulated genes following DENV infection, among which 30 genes (e.g., *Tceb1*, *Phf3*, and *Ptcd*) were successfully corrected to preinfection (control) levels, while the remaining genes (e.g., *Apcs*, *Cyp4a10*, and *Aqp1*) did not show evidence of normalization. The normalization in the expression levels of the DENV-induced dysregulated genes in the DVI-SCT group showed that stem cells are able to modulate gene dysregulation caused by dengue infection. Stem cells treatment corrected up- or downregulated genes in the infection fully (or partially) to the normalized gene level, suggesting the potential of stem cells in treating DENV infection at the genetic level.

To determine the function of a particular differentially expressed gene, all differentially expressed genes were mapped into a Gene Ontology (GO) database. Twelve genes involved in the cell cycle pathway and regulation were selected for analysis (Figure 3Ca). Of the 12 selected genes, 6 showed upregulation, while the other 6 showed downregulation upon DENV2 infection. All the gene (both up- and downregulated) expression levels were normalized following treatment with stem cells except for three of the upregulated genes. Upregulation of the gene Gsk3b, which is involved in metabolic pathways, including inflammation, stress, mitochondrial dysfunction, and apoptosis (Jope et al., 2007), was adjusted to reflect control levels. The phosphatidylinositol-3-kinase/protein kinase B (PI3K-Akt) pathway gene subunit, *Pik3r1*, which plays a key role in cell cycle, apoptosis, and DNA repair (Franke, 2008), was continuously upregulated upon stem cell treatment to ensure increased platelet proliferation and white blood cell count, countering the apoptosis and thrombocytopenia seen in dengue infection. This cements the hypothesis that both HSCs and EPCs play a vital role in ensuring regulation of cell cycle pathways and counteracting the negative effects of dengue infection.

In addition, genes related to apoptosis were also analyzed in the obtained NGS data (Figure 3Cb). The results showed that the all the genes related to apoptotic pathways (Casp2, 3, 4, and 6 and 8ap2) that were dysregulated in dengue infection were corrected to a level similar to that in control samples following stem cell treatment. This shows that the widespread apoptosis caused by dengue infection was reduced and regulated by stem cell treatment, ensuring correction of gene expression and a decrease in swelling and the size of hepatocytes in addition to reduced apoptosis and sinusoidal spaces in hepatocytes (Paes et al., 2005; Sakinah et al., 2017). Taken together, the NGS data further validated the histopathological findings of this study, thus suggesting that stem cell treatment is a superior treatment option for dengue infection.

In the recent years, the incidence of dengue has continued to increase, but there is currently no rapid and effective treatment available. This study demonstrates stem cell therapy as a promising management to reduce the global burden of dengue. Since this is the first study done to treat dengue infection with stem cells, we faced several limitations such as very little research is written on this subject to use as a reference and inadequate blood samples to perform additional quantitative test for validation. Hence, this study could be an exploratory study that lay the groundwork for more complete research in the future. Our study recommends a more detailed study on host responses, mainly the interferon (IFN) signaling to stem cell therapy, which would provide the mechanism underlying the host–pathogen interaction A robust study is required to provide more evidence for the effectiveness of this treatment including observing the effect of the treatment in other organs such as the spleen, kidney, and brain, identifying the virus load and gamma-GGT levels and elucidating the antiviral genes in tissues and serums, exploring the presence of antibodies and antigens of the virus by RT-PCR, characterizing various proinflammatory cytokines that are involved in dengue infection, and using TUNEL assay to analyze apoptosis.

## Materials and Methods

### Mouse Model of Dengue Infection and Stem Cell Therapy

This study was carried out in the animal house, Faculty of Veterinary Medicine, UPM. All *in vivo* procedures were performed with the ethical approval of the IACUC, Universiti Putra Malaysia (ref. no. UPM/IACUC/AUP-R017/2015). Seventy-two 8-week-old male BALB/c mice (20–25 g) were acclimated to the environment for 1 week and divided into three groups: control, DVI, and DVI-SCT groups (24 mice each group). EMEM (control group) and DENV 2 (1.25 × 10^5^ FFU/ml) (DVI and DVI-SCT groups) were injected intraperitoneally according to different groups for 2 consecutive days (500 μl/day). Thereafter, the control group and the DVI group were intravenously injected with PBS for 3 consecutive days. The DVI-SCT group was injected with specific stem cells [HSCs (2.5 × 10^5^ cells per mouse) and EPCs (1 × 10^6^ cells per mouse)] and growth factors (1 μg/kg/day for 3 days). After 2 weeks, PBS (control and DVI groups) and specific stem cells [HSCs (2.5 × 10^5^ cells per mouse) and EPCs (1 × 10^6^ cells per mouse)] and growth factor (1 μg/kg/day for 1 day) were intravenously injected in 100 μl according to different groups. Blood of each group was collected for hematological analysis (scil Vet abc^TM^, Horiba, Germany) on 5, 10, 15, and 21 dpi and at the above four time points; six mice from each group were sacrificed and collected for histological examination, DENV immunohistochemical staining, and differentially expressed genes and transcriptomics analysis.

### Propagation of DENV 2

*Aedes albopictus* clone (C6/36) cells (ATCC^®^ CRL-1660^TM^, United States) were grown in a T25 flask, and the cells were subcultured until an adequate number of cells were obtained for DENV 2 viral inoculation and propagation. The procedure was performed following that described by Ip and Liao (2010) with different culture medium, as suggested by ATCC [Eagle’s minimum essential media (EMEM, Biowest, Riverside, CA, United States) supplemented with 10% fetal bovine serum (FBS, Biowest) and 1% penicillin-streptomycin (Biowest)] (Ip and Liao, 2010). When the C6/36 cells reached 80% confluency at passage 3, 1 ml of DENV 2, a clinical isolate obtained from the virology laboratory at UPM, was inoculated into the confluent cells. The flask containing DENV 2 was incubated at 25°C for 1 h with 10 rpm agitation. After that, 5 ml of EMEM supplemented with 2% FBS was added, and the flask was incubated at 28°C for 8 days at a multiplicity of infection (MOI) of 0.1. The cytopathic effects (CPEs) were observed every day. After 8 days of incubation, the virus was isolated using a rapid freeze–thaw technique. This procedure was repeated for up to 10 passages to obtain a higher virus titer (Mota and Rico-Hesse, 2011; Martinez-Gutierrez et al., 2014). For confirmation of the DENV serotype, an immunofluorescence assay was performed using antibodies directed against all four DV serotypes following a protocol reported by Medina et al. (2012). Briefly, an immunofluorescence assay was performed using cultured infected cells that were fixed on a multiwell slide (Supplementary Figure 1). The cells were stained with DV serotype-specific monoclonal antibodies (mAbs, Merck Millipore, Darmstadt, Germany) and then with fluorescein isothiocyanate (FITC)-conjugated antimouse antibody (Merck Millipore). The slide was later examined under a fluorescence light microscope at × 100 magnification for observation of positive cells. The culture supernatant containing DENV 2 was collected and filtered through a 0.22 μM filter. A foci forming assay was also performed at passage 10. Vero cells were inoculated with serially diluted DENV 2, followed by staining with monoclonal anti-DENV antibodies, secondary antibodies conjugated with enzymes, and finally with the metal enhanced 3,3′-diaminobenzidine (DAB) substrate. The dark brown foci were visualized and counted under a stereo microscope on the fifth dpi. The virus titer was obtained (1.25 × 10^5^ FFU/ml) through viral quantitation using the following formula: virus titer, FFU/ml (foci forming unit per milliliter) = average of foci/dilution factor × virus inoculums volume (Zandi et al., 2012). The virus stock is stored at −80°C for subsequent infection.

### Preparation of Hematopoietic Stem Cells

Hematopoietic stem cells were isolated from bone marrow cells of five 8-week-old BALB/c mice. Briefly, tibias and femurs were obtained and cut open to expose the bone marrow cavity. The bone marrow cavity was carefully punctured using a 26G needle, and the bone marrow was flushed out with Dulbecco’s modified Eagle’s medium (DMEM, Gibco) supplemented with 2% FBS (Gibco, Waltham, MA, United States) until there was no visible reddish line in the bone. Cells were placed into a 50 ml tube for isolation of CD117-positive cells (HSCs). The CD117-positive cells were isolated using a CD117-positive selection kit (Easysep^®^, STEMCELL Technologies, Singapore) with an EasySep^®^ magnet (Kroeger et al., 2009).

Cell suspension with a concentration of 1 × 10^7^ cells per 100 μl was prepared in PBS (Invitrogen, Carlsbad, CA, United States) with 2% FBS. Cells were incubated in CD117 PE labeling reagent (50 μl/ml) with mixing for 15 min at room temperature. PE selection cocktail (70 μl/ml) was applied for 15 min at room temperature. Next, the cells were mixed with 50 μl/ml nanoparticles and incubated at room temperature for 10 min. The cell suspension was brought to a total volume of 2.5 ml and set aside for 5 min. The EasySep^®^ magnet was inverted in one continuous motion to pour off the supernatant. This washing method was repeated four times to avoid cell contamination. Finally, the tube was removed from the magnet, and the cells were resuspended in DMEM supplemented with 15% FBS for expansion (Dudeck et al., 2011). CD 117-positive cells were cultured in high-glucose DMEM supplemented with 15% FBS, 1% penicillin-streptomycin (Biowest), 100 ng/ml murine stem cell factor (SCF) (BioVision, Milpitas, CA, United States), 6 ng/ml murine IL-3 (BioVision), and 10 ng/ml human IL-6 (BioVision). The medium was replaced with fresh medium every 3 days until the cells reached confluence, and cell culture was continued until the cells reached passage 3 (Sekulovic et al., 2008). The expanded cells were further characterized for HSCs surface markers [with APC-conjugated CD133, FITC-conjugated CD34, FITC-conjugated CD135, and PE-conjugated CD117 antibodies (eBioscience, San Diego, CA, United States)] using flow cytometry (FACS Aria III cell sorter) (Frascoli and Proietti, 2012).

### Preparation of Endothelial Progenitor Cells

BALB/c mouse bone marrow-derived endothelial progenitor cells were purchased from Cell Biologics, Inc. (Cell Biologics, Chicago, IL, United States). The cell revival and culture protocols were carried out according to the company instructions. The cells in the cryovial were quickly thawed in a 37°C water bath for <1 min, resuspended in 7 ml of prewarmed Cell Biologics cell culture medium, and added to a fresh tube. After centrifugation, the cells were resuspended in cell culture medium supplemented with 10% FBS, hydrocortisone, VEGF, EGF, endothelial cell growth factor (ECGS), heparin, L-glutamine, and antibiotic–antimycotic solution. Cells were seeded into a gelatin-coated flask and incubated in a 5% CO_2_ incubator at 37°C. Culture medium was changed 3 days after the culture start time and then every 2 days (Kalka et al., 2000). To identify the characteristics of BM-EPCs, the cells were analyzed with a FACS Aria III cell sorter using APC-conjugated CD133, PE-conjugated CD309, FITC-conjugated CD34, FITC-conjugated CD 135, and PE-conjugated CD117 antibodies (eBioscience) (Tian et al., 2019).

Flow cytometric analysis results showed that the majority of the adherent cells displayed both the morphological and qualitative properties of EPCs (Supplementary Figure 2). The cell population positive for CD34 was selected. From the singlet cell population, 72.1% expressed the marker CD34. Among the CD34-positive cells, 71.4% expressed the marker CD133, 89.9% expressed the marker CD309, and 93.9% expressed the marker CD117.

### Hematological and Biochemical Assays

Blood was collected via cardiac puncture on the 5th, 10th, 15th, and 21st dpi (Supplementary Figure 3). The mice were anesthetized with ketamine (100 mg/kg, Ilium) and xylazine (10 mg/kg, Ilium) using a previously described process. Blood samples of 400 μl were sent for each analysis using an automated hematological and biochemical analyzer.

### Histopathology

After the mice were euthanized, the livers were collected and stored in 10% formalin. The liver tissue was randomly chosen for sections processing. Microscopic slides were obtained after preparation of wax blocks and staining with hematoxylin and eosin (Leica, IL, United States). Tissue sections were observed under a microscope for differential cell counts. The histopathological sections from each mouse were analyzed quantitatively for the different cell populations percentage, including Kupffer cells, apoptotic cells, mononucleated hepatocytes, and binucleated hepatocytes. The sections were observed under a Nikon Eclipse 50i light microscope, and the image was captured with a Nikon DS-FI1 digital camera. Each slide contained three sections obtained at 15-μM intervals, and random six different areas were examined with 3 × 10^4^ μm overlaid grid. The cells were scored with NIS elements D image analysis software (Nikon, New York, NY, United States). Each (mouse) slide was examined with six different areas of 18 × 10^4^ μm^2^ and each group examined for 54 × 10^4^ μm^2^. The hemorrhagic areas were observed in 20 magnifications (to include a wider area of 216 × 10^4^ μm^2^ per group). All the mean values were analyzed using one-way ANOVA, IBM SPSS statistics version 23, and represented in bar chart.

### Immunohistochemistry for DENV 2 Detection

Paraffin-embedded tissues were sectioned at a thickness of 5 μM and prepared on a slide. The mounted sections were deparaffinized at 60°C for 2 h and then immersed into xylene (HmbG Bendosen, Kuala Lumpur, Malaysia), with two changes at 5 min each. The slide was then transferred into 100% ethanol (Fisher Scientific, MA, United States) and incubated for 5 min, followed by 70% ethanol for 5 min. Next, the slides were immersed into Tris-EDTA buffer (pH 9.0) for 45 min at 95°C for antigen retrieval. After that, the slides were brought to room temperature, allowed to cool for 20 min, washed with PBS twice for 2 min, and permeabilized with 0.25% Triton X-100 (Thermo Scientific) in PBS. The slides were then rinsed with PBS, and excess water was blotted from the slide holder for 20 min at 27°C. Mouse anti-DENV primary antibodies (ab480914, Acris GmbH, Luzern, Switzerland) were added to the slides at 1:200 dilution. Slides were incubated overnight at 4°C. The next day, the slides were washed with PBS at room temperature and further incubated for 20 min at 37°C. After that, diluted goat antimouse Alexa Flour 488-conjugated secondary antibody (1:500, BioLegend, San Diego, CA, United States) was added and incubated with each section for 1 h in a 37°C incubator. The slide was further washed with PBS and dried for 15 min at 37°C. The slides were then counterstained with 4′,6-diamidino-2-phenylindole (DAPI) at a 1:1,000 dilution for 5 min, rinsed and covered with mounting fluid. The slides were observed under a fluorescence light microscope at × 200 magnification for any positive expression for DENV.

### Transmission Electron Microscopy

The desired area of liver tissues was selected and cut into 1 mm × 1 mm slices. The tissue was immediately fixed in 3% glutaraldehyde in 0.1% phosphate buffer for 4 h, rinsed with phosphate buffer three times for 10 min each time, and post-fixed with 0.1% osmium tetroxide in 0.1% phosphate buffer for 4 h. The tissue was further processed for en bloc staining with 3% aqueous uranyl acetate in the dark for 1 h at room temperature. After staining, the specimen was washed, dehydrated, and embedded in resin in a beam capsule. The tissue block was polymerized for 78 h, sectioned into 0.5–1-μm sections and dried. These semithin sections were stained with toluidine blue for 2–5 min to select the area of interest. The selected section was trimmed, and ultrathin 70 nm thick sections were cut with special diamond knives and individually transferred onto a copper grid. The sections were stained with uranyl acetate and lead citrate before viewing under a transmission electron microscope.

### RNA Preparation and Sequencing for Transcriptomic Analysis

RNA sequencing of *de novo* infected livers was performed with an MiSeq next-generation sequencer (Illumina, Inc., San Diego, CA, United States). Total RNA was extracted from liver tissues using a Qiagen RNeasy Mini kit with in-column DNase treatment as per the manufacturer’s instructions (Sessions et al., 2013). RNA concentration and purity were assessed using a NanoDrop 2000c spectrophotometer (NanoDrop Technologies, Wilmington, DE, United States), and RNA integrity was analyzed on a Bioanalyzer 2100 (Agilent Technologies, Santa Clara, CA, United States). A NEBNext Ultra RNA library prep kit for Illumina (New England Biolabs, Ipswich, MA, United States) was used to prepare complementary DNA (cDNA) libraries for RNA sequencing as per the manufacturer’s instructions. The fragment size, purity, and quantities of the prepared libraries were determined using the Bioanalyzer 2100 (Agilent Technologies). The libraries were then sequenced using a MiSeq reagent kit V3 (Illumina).

### Gene Expression (Transcriptomic Analysis)

Transcriptome assembly and analysis were completed using Tuxedo software. The libraries sequenced were mapped to the updated genomic annotation file of the mouse genome (*Mus musculus*, NCBI ref. database: GCF_000001635.26_GRCm38.p6_genomic.gff) using Tophat v2.0.9 (Trapnell et al., 2009; Kim et al., 2013). Transcripts were assembled following conformation of reads quality control via the FASTQ format and normalized and quantified by Cufflinks 2.0.2 analysis (Trapnell et al., 2010). Isoforms assembled by Cufflinks from all the sample groups were further merged to create a single transcriptome annotation file in gtf using Cuffmerge. The results were analyzed using Cuffdiff to identify differentially expressed genes and transcripts. The relative expression of genes was determined based on FPKM (total fragments per kilobase of exon per million mapped reads) values. The results obtained were used to generate density and scatter volcano plots using CummeRbund software. Heat maps showing the relative expression of the differentially expressed genes following treatment with stem cells were also generated utilizing the Morpheus heat map generator^1^.

### Data Analysis

The mean values and significant differences were calculated via one-way ANOVA using version 23 IBM SPSS statistics software.

## Data Availability Statement

The data presented in the study are deposited in the (GEO) repository, accession number (GSE175707).

## Ethics Statement

The animal study was reviewed and approved by the Ethical Committee of the IACUC, Universiti Putra Malaysia (ref. no. UPM/IACUC/AUP-R017/2015).

## Author Contributions

SK designed the research study, supervised and validated the experiments, edited and finalized the manuscript. SS experimented and contributed in the preparation of stem cells, preparation of DENV2, animal studies, hematological and biochemical assays, histopathology, immunohistochemistry for DENV2 detection, transmission electron microscopy, RNA preparation, data collection and analysis, and manuscript writeup. SP contributed in transmission electron microscopy, hematological and biochemical assays. RM and FB contributed in transcriptomic analysis. PM supervised the preparation of stem cells and edited the manuscript. ST and ZS analyzed data, drafted the manuscript, and prepared the figures. H-YC helped in the propagation of DENV2. RH, GH, BA, CX, NJ, JT, XW, MM, AS, and AH reviewed the manuscript. All authors contributed to the article and approved the submitted version.

## Conflict of Interest

The authors declare that the research was conducted in the absence of any commercial or financial relationships that could be construed as a potential conflict of interest. The reviewer RR declared a shared affiliation, with no collaboration, with one of the authors, AH, to the handling editor at the time of the review.

## Footnotes

1
https://software.broadinstitute.org/morpheus/

## References

[B1] Akashi K. Traver D. Miyamoto T. Weissman I. L. (2000). A clonogenic common myeloid progenitor that gives rise to all myeloid lineages. *Nature* 404 193–197. 10.1038/35004599 10724173

[B2] Baldridge M. T. King K. Y. Goodell M. A. (2011). Inflammatory signals regulate hematopoietic stem cells. *Trends Immunol.* 32 57–65. 10.1016/j.it.2010.12.003 21233016 PMC3042730

[B3] Baldridge M. T. King K. Y. Boles N. C. Weksberg D. C. Goodell M. A. (2010). Quiescent haematopoietic stem cells are activated by IFN-gamma in response to chronic infection. *Nature* 465 793–797. 10.1038/nature09135 20535209 PMC2935898

[B4] Barth O. M. Barreto D. F. Paes M. V. Takiya C. M. Pinhão A. T. Schatzmayr H. G. (2006). Morphological studies in a model for dengue-2 virus infection in mice. *Mem. Inst. Oswaldo Cruz* 101 905–915. 10.1590/s0074-02762006000800014 17293987

[B5] Basilio-de-Oliveira C. A. Aguiar G. R. Baldanza M. S. Barth O. M. Eyer-Silva W. A. Paes M. V. (2005). Pathologic study of a fatal case of dengue-3 virus infection in Rio de Janeiro, Brazil. *Braz. J. Infect. Dis.* 9 341–347. 10.1590/s1413-86702005000400012 16270128

[B6] Bhatt S. Gething P. Brady O. Messina J. Farlow A. W. Moyes C. L. (2013). The global distribution and burden of dengue. *Nature* 496:504. 10.1038/nature12060 PMC3651993 23563266

[B7] Boelig M. M. Kim A. G. Stratigis J. D. McClain L. E. Li H. Flake A. W. (2016). The Intravenous Route of Injection Optimizes Engraftment and Survival in the Murine Model of In Utero Hematopoietic Cell Transplantation. *Biol. Blood Marrow Transplant.* 22:991e999. 10.1016/j.bbmt.2016.01.017 26797401

[B8] Brudecki L. Ferguson D. A. Yin D. Lesage G. D. McCall C. E. El Gazzar M. (2012). Hematopoietic stem-progenitor cells restore immunoreactivity and improve survival in late sepsis. *Infect. Immun.* 80 602–611. 10.1128/iai.05480-11 22144495 PMC3264285

[B9] Dudeck A. Suender C. A. Kostka S. L. von Stebut E. Maurer M. (2011). Mast cells promote Th1 and Th17 responses by modulating dendritic cell maturation and function. *Eur. J. Immunol.* 41 1883–1893. 10.1002/eji.201040994 21491417

[B10] Franca R. F. Zucoloto S. da Fonseca B. A. (2010). A BALB/c mouse model shows that liver involvement in dengue disease is immune-mediated. *Exp. Mol. Pathol.* 89 321–326. 10.1016/j.yexmp.2010.07.007 20673760

[B11] Franke T. (2008). PI3K/Akt: getting it right matters. *Oncogene* 50:6473. 10.1038/onc.2008.313 18955974

[B12] Frascoli M. Proietti M. (2012). F Grassi F. Phenotypic analysis and isolation of murine hematopoietic stem cells and lineage-committed progenitors. *J. Vis. Exp.* 65:e3736. 10.3791/3736 PMC3471276 22805770

[B13] Frias-Staheli N. Dorner M. Marukian S. Billerbeck E. Labitt R. N. Rice C. M. (2014). Utility of Humanized BLT Mice for Analysis of Dengue Virus Infection and Antiviral Drug Testing. *J. Virol.* 88 2205–2218. 10.1128/jvi.03085-13 24335303 PMC3911540

[B14] Fujii H. Hirose T. Oe S. Yasuchika K. Azuma H. Fujikawa T. (2002). Contribution of bone marrow cells to liver regeneration after partial hepatectomy in mice. *J. Hepatol.* 36 653–659. 10.1016/s0168-8278(02)00043-0 11983449

[B15] Geffner L. F. Santacruz P. Izurieta M. Flor L. Maldonado B. Auad A. H. (2008). Administration of autologous bone marrow stem cells into spinal cord injury patients via multiple routes is safe and improves their quality of life: comprehensive case study. *Cell Transplant.* 17 1277–1293. 10.3727/096368908787648074 19364066

[B16] Ip P.-P. Liao F. (2010). Resistance to dengue virus infection in mice is potentiated by CXCL10 and is independent of CXCL10-mediated leukocyte recruitment. *J. Immunol.* 184 5705–5714. 10.4049/jimmunol.0903484 20400703

[B17] Jiang X. Chen Y. Wei H. Sun R. Tian Z. (2013). Characterizing the lymphopoietic kinetics and features of hematopoietic progenitors contained in the adult murine liver in vivo. *PLoS One* 8:e76762. 10.1371/journal.pone.0076762 24130788 PMC3793923

[B18] Jope R. S. Yuskaitis C. J. Beurel E. (2007). Glycogen Synthase Kinase-3 (GSK3): inflammation, Diseases, and Therapeutics. *Neurochem. Res.* 32 577–595. 10.1007/s11064-006-9128-5 16944320 PMC1970866

[B19] Kalka C. Masuda H. Takahashi T. (2000). Transplantation of ex vivo expanded endothelial progenitor cells for therapeutic neovascularization. *Proc. Natl. Acad. Sci. U. S. A.* 97 3422–3427. 10.1073/pnas.97.7.3422 10725398 PMC16255

[B20] Kallis Y. N. Alison M. R. Forbes S. J. (2007). Bone marrow stem cells and liver disease. *Gut* 56 716–724. 10.1136/gut.2006.098442 17145739 PMC1942133

[B21] Kim S. Park J. Kim J. Lee J. Bang B. (2013). RNAseq-based transcriptome analysis of Burkholderia glumae quorum sensing. *Plant Pathol. J.* 29 249–259. 10.5423/ppj.oa.04.2013.0044 25288952 PMC4174805

[B22] Kroeger K. Collins M. Ugozzoli L. (2009). The preparation of primary hematopoietic cell cultures from murine bone marrow for electroporation. *J. Vis. Exp.* 23:1026. 10.3791/1026 PMC2763291 19229174

[B23] Kuo C. H. Tai D. I. Chang-Chien C. S. Lan C. K. Chiou S. S. Liaw Y. F. (1992). Liver biochemical tests and dengue fever. *Am. J. Trop. Med. Hyg.* 47 265–270. 1355950 10.4269/ajtmh.1992.47.265

[B24] Kuruvilla J. G. Troyer R. M. Devi S. Akkina R. (2007). Dengue virus infection and immune response in humanized RAG2 (-/-) gamma(c)(-/-) (RAG-hu) mice. *Virology* 369 143–152. 10.1016/j.virol.2007.06.005 17707071

[B25] Lagasse E. Connors H. Al-Dhalimy H. Reitsma M. Dohse M. Osborne L. (2000). Purified hematopoietic stem cells can differentiate into hepatocytes in vivo. *Nat. Med.* 6 1229–1234. 10.1038/81326 11062533

[B26] Lawn S. D. Tilley R. Lloyd G. Finlayson C. Tolley H. Newman P. (2003). Dengue hemorrhagic fever with fulminant hepatic failure in an immigrant returning to Bangladesh. *Clin. Infect. Dis.* 37 e1–e4. 10.1086/375601 12830429

[B27] Low J. S. Y. Wu K. X. Chen K. C. Ng M. M.-L. Chu J. J. H. (2011). Narasin, a novel antiviral compound that blocks dengue virus protein expression. *Antivir. Ther.* 16 1203–1218. 10.3851/imp1884 22155902

[B28] Lozito T. Kolf C. Tuan R. S. (2008). “Microenvironmental regulation of adult mesenchymal stem cells,” in *Regulatory Networks in Stem Cells*, eds Rajasekhar V. K. Vemuri M. C. (Totowa: Humana Press).

[B29] Lozito T. Kolf C. Tuan R. S. (2009). “Microenvironmental regulation of adult mesenchymal stem cells,” in *Regulatory Networks in Stem Cells*, eds Rajasekhar V. K. Vemuri M. C. (Totowa: Humana Press), 185–210. 10.1007/978-1-60327-227-8_17

[B30] Martinez-Gutierrez M. Correa-Londoño L. A. Castellanos J. E. Gallego-Gómez J. C. Osorio J. E. (2014). Lovastatin delays infection and increases survival rates in AG129 mice infected with dengue virus serotype 2. *PLoS One* 9:e87412. 10.1371/journal.pone.0087412 24586275 PMC3931612

[B31] McAllister S. Medina R. O’Neill C. Stitt A. W. (2013). Characterisation and therapeutic potential of endothelial progenitor cells. *Lancet* 381:S73.

[B32] Medina F. Medina J. F. Colon C. Vergne E. Santiago G. A. Munoz-Jordan J. L. (2012). Dengue virus: isolation, propagation, quantification, and storage. *Curr. Protoc. Microbiol.* 15:15D.2. 10.1002/9780471729259.mc15d02s27 23184594

[B33] Mota J. Rico-Hesse R. (2011). Dengue virus tropism in humanized mice recapitulates human dengue fever. *PLoS One* 6:e20762. 10.1371/journal.pone.0020762 21695193 PMC3112147

[B34] Nagy R. D. Tsai B. M. Wang M. Markel T. A. Brown J. W. Meldrum D. R. (2005). Stem cell transplantation as a therapeutic approach to organ failure. *J. Surg. Res.* 129 152–160. 10.1016/j.jss.2005.04.016 16045936

[B35] Paes M. V. Pinhão A. T. Barreto D. F. Costa S. M. Oliveira M. P. Nogueira A. C. (2005). Liver injury and viremia in mice infected with dengue-2 virus. *Virology* 338 236–246. 10.1016/j.virol.2005.04.042 15961136

[B36] Paes M. Lenzi H. Nogueira A. Nuovo G. J. Pinhao A. T. Mota E. M. (2009). Hepatic damage associated with dengue-2 virus replication in liver cells of BALB/c mice. *Lab. Invest.* 89:1140. 10.1038/labinvest.2009.83 19721415

[B37] Pascutti M. F. Erkelens M. N. Nolte M. A. (2016). Impact of Viral Infections on Hematopoiesis: from Beneficial to Detrimental Effects on Bone Marrow Output. *Front. Immunol.* 7:364. 10.3389/fimmu.2016.00364 27695457 PMC5025449

[B38] Purwati Sumorejo Purnama D. S. Miatmoko A. Nasronudin N. Dinaryanti A. Karsari D. (2020). The Potential of Hematopoietic Stem Cells (Hsc) Against Sars-Cov-2 (Covid-19) With Virus Isolates From Indonesia (In Vitro Study). research squares. preprint.

[B39] Ruhnke M. Nussler A. K. Ungefroren H. Hengstler J. G. Kremer B. Hoeckh W. (2005). Human monocyte-derived neohepatocytes: a promising alternative to primary human hepatocytes for autologous cell therapy. *Transplantation* 79 1097–1103. 15880050 10.1097/01.tp.0000157362.91322.82

[B40] Sakinah S. Priya S. Kumari S. Amira F. Poorani K. Alsaeedy H. (2017). Impact of dengue virus (serotype DENV-2) infection on liver of BALB/c mice: a histopathological analysis. *Tissue Cell* 49 86–94. 10.1016/j.tice.2016.11.005 28034555

[B41] Salazar Vázquez B. Y. Cabrales P. Tsai A. G. Johnson P. C. Intaglietta M. (2008). Lowering of blood pressure by increasing hematocrit with non nitric oxide scavenging red blood cells. *Am. J. Respir. Cell Mol. Biol.* 38 135–142. 10.1165/rcmb.2007-0081OC 17709601 PMC2214674

[B42] Salter A. B. Meadows S. K. Muramoto G. G. Himburg H. Doan P. Daher P. (2009). Endothelial progenitor cell infusion induces hematopoietic stem cell reconstitution in vivo. *Blood* 113 2104–2107. 10.1182/blood-2008-06-162941 19141867 PMC2651019

[B43] Samanta J. Sharma V. (2015). Dengue and its effects on liver. *World J. Clin. Cases* 3 125–131. 10.12998/wjcc.v3.i2.125 25685758 PMC4317605

[B44] Sekulovic S. Imren S. Humphries K. (2008). High level in vitro expansion of murine hematopoietic stem cells. *Curr. Protoc. Stem Cell Biol.* 4:2A.7. 10.1002/9780470151808.sc02a07s4 18770636

[B45] Seneviratne S. L. Malavige G. N. de Silva H. J. (2006). Pathogenesis of liver involvement during dengue viral infections. *Trans. R. Soc. Trop. Med. Hyg.* 100 608–614. 10.1016/j.trstmh.2005.10.007 16483623

[B46] Sessions O. M. Tan Y. Goh K. C. Liu Y. Tan P. Rozen S. (2013). Host Cell Transcriptome Profile during Wild-Type and Attenuated Dengue Virus Infection. *PLoS Negl. Trop. Dis.* 7:e2107. 10.1371/journal.pntd.0002107 23516652 PMC3597485

[B47] Sreekanth G. P. Chuncharunee A. Sirimontaporn A. Panaampon J. Noisakran S. Yenchitsomanus P.-T. (2016). SB203580 Modulates p38 MAPK Signaling and Dengue VirusInduced Liver Injury by Reducing MAPKAPK2, HSP27, and ATF2 Phosphorylation. *PLoS One* 11:e0149486. 10.1371/journal.pone.0149486 26901653 PMC4764010

[B48] Sreekanth G. P. Chuncharunee A. Sirimontaporn A. Panaampon J. Srisawat C. Morchang A. (2014). Role of ERK1/2 signaling in dengue virus-induced liver injury. *Vir. Res.* 188 15–26. 10.1016/j.virusres.2014.03.025 24704674

[B49] Sreekanth G. P. Panaampon J. Suttitheptumrong A. Chuncharunee A. Bootkunha J. Yenchitsomanus P. (2019). Drug repurposing of N-acetyl cysteine as antiviral against dengue virus infection. *Antivir. Res.* 166 42–55. 10.1016/j.antiviral.2019.03.011 30928439

[B50] Tan G. K. Ng J. K. Lim A. H. Yeo K. P. Angeli V. Alonso S. (2011). Subcutaneous infection with non-mouse adapted Dengue virus D2Y98P strain induces systemic vascular leakage in AG129 mice. *Ann. Acad. Med. Singap.* 40 523–532. 22294063

[B51] Taniguchi E. Kin M. Torimura T. Nakamura T. Kumemura H. Hanada S. (2006). Endothelial progenitor cell transplantation improves the survival following liver injury in mice. *Gastroenterology* 130 521–531. 10.1053/j.gastro.2005.10.050 16472604

[B52] Tian F. Liang P. Li L. (2019). Inhibition of endothelial progenitor cell differentiation by VEGI. *Blood* 113 5352–5360. 10.1182/blood-2008-08-173773 19329781 PMC2686197

[B53] Trapnell C. Pachter L. Salzberg S. (2009). TopHat: discovering splice junctions with RNA-Seq. *Bioinformatics* 25 1105–1111. 10.1093/bioinformatics/btp120 19289445 PMC2672628

[B54] Trapnell C. Williams B. Pertea G. Mortazavi A. (2010). Transcript assembly and quantification by RNA-Seq reveals unannotated transcripts and isoform switching during cell differentiation. *Nat. Biotechnol.* 28 511–515. 10.1038/nbt.1621 20436464 PMC3146043

[B55] Woolthuis C. M. Park C. Y. (2016). Hematopoietic stem/progenitor cell commitment to the megakaryocyte lineage. *Blood* 127 1242–1248. 10.1182/blood-2015-07-607945 26787736 PMC5003506

[B56] World Health Organization (WHO). (2009). *Dengue Guidelines for Diagnosis, Treatment, Prevention and Control: New Edition.* Geneva: WHO Press. 23762963

[B57] Yang Z. Cai X. Xu A. Xu F. Liang Q. (2015). Bone marrow stromal cell transplantation through tail vein injection promotes angiogenesis and vascular endothelial growth factor expression in cerebral infarct area in rats. *Cytotherapy* 17 1200–1212. 10.1016/j.jcyt.2015.06.005 26276003

[B58] Zandi K. Lani R. Wong P. Teoh B. Sam S. Johari J. (2012). Flavone enhances dengue virus type-2 (NGC strain) infectivity and replication in Vero cells. *Molecules* 17 2437–2445. 10.3390/molecules17032437 22374315 PMC6268591

[B59] Zellweger R. M. Cano J. Mangeas M. Taglioni F. Mercier A. Despinoy M. (2017). Socioeconomic and environmental determinants of dengue transmission in an urban setting: an ecological study in Nouméa, New Caledonia. *PLoS Negl. Trop. Dis.* 11:e0005471. 10.1371/journal.pntd.0005471 28369149 PMC5395238

[B60] Zhang Y. Bai X.-F. Huang C.-X. (2003). Hepatic stem cells: existence and origin. *World J. Gastroenterol.* 9 201–204. 10.3748/wjg.v9.i2.201 12532431 PMC4611311

[B61] Zheng J. Song C. Zhang C. C. (2011). A new chapter: hematopoietic stem cells are direct players in immunity. *Cell Biosci.* 1:33. 10.1186/2045-3701-1-33 21978817 PMC3198676

