# Corrigendum: Stem Cell Therapy in Dengue Virus-Infected BALB/C Mice Improves Hepatic Injury

**DOI:** 10.3389/fcell.2021.800659

**Published:** 2022-02-01

**Authors:** S. Sakinah, Sivan Padma Priya, Pooi Ling Mok, Rusheni Munisvaradass, Seoh Wei Teh, Zhong Sun, Badr Alzahrani, Faizal Abu Bakar, Hui-Yee Chee, Rukman Awang Hamat, Guozhong He, Chenglong Xiong, Narcisse Joseph, Jia Bei Tong, Xiaoyun Wu, Mahendran Maniam, Antony V. Samrot, Akon Higuchi, S. Suresh Kumar

**Affiliations:** ^1^ Department of Medical Microbiology, Faculty of Medicine and Health Sciences, Universiti Putra Malaysia, Seri Kembangan, Malaysia; ^2^ Department of Clinical Laboratory Sciences, College of Applied Medical Sciences, Jouf University, Sakakah, Saudi Arabia; ^3^ Department of Biomedical Sciences, Faculty of Medicine and Health Sciences, Universiti Putra Malaysia, Seri Kembangan, Malaysia; ^4^ Bioinformatics and Computational Biology, Malaysia Genome Institute, National Institute of Biotechnology Malaysia (NIBM), Kajang, Malaysia; ^5^ Institute of Health, Kunming Medical University, Kunming, China; ^6^ Department of Medical Microbiology, School of Public Health, Fudan University, Shanghai, China; ^7^ First Affiliated Hospital of Baotou Medical College, Inner Mongolia University of Science and Technology, Baotou, China; ^8^ Sultan Idris Education University, Tanjong Malim, Malaysia; ^9^ School of Bioscience, Faculty of Medicine, Bioscience and Nursing, MAHSA University, Jenjarom, Malaysia; ^10^ Department of Chemical and Materials Engineering, National Central University, Taoyuan City, Taiwan; ^11^ R&D Center for Membrane Technology, Chung Yuan Christian University, Taoyuan City, Taiwan; ^12^ Centre for Materials Engineering and Regenerative Medicine, Bharath Institute of Higher Education and Research, Chennai, India

**Keywords:** dengue infection, stem cell therapy, DENV 2, next-generation sequencing, hepatology

In the published article, there was an error in **affiliations 8, 9, 10, 11, 12**. Instead of “^8^ School of Bioscience, Faculty of Medicine, Bioscience and Nursing, MAHSA University, Jenjarom, Malaysia, ^9^ Department of Chemical and Materials Engineering, National Central University, Taoyuan City, Taiwan, ^10^ R&D Center for Membrane Technology, Chung Yuan Christian University, Taoyuan City, Taiwan, ^11^ Centre for Materials Engineering and Regenerative Medicine, Bharath Institute of Higher Education and Research, Chennai, India.” It should be “^8^ Sultan Idris Education University, Tanjong Malim, Malaysia, ^9^ School of Bioscience, Faculty of Medicine, Bioscience and Nursing, MAHSA University, Jenjarom, Malaysia, ^10^ Department of Chemical and Materials Engineering, National Central University, Taoyuan City, Taiwan, ^11^ R&D Center for Membrane Technology, Chung Yuan Christian University, Taoyuan City, Taiwan, ^12^ Centre for Materials Engineering and Regenerative Medicine, Bharath Institute of Higher Education and Research, Chennai, India.”

In the published article, there was an error regarding the **affiliations for Mahendran Maniam, Antony V. Samrot, Akon Higuchi and S. Suresh Kumar**. Instead of having affiliations “Mahendran Maniam^7^, Antony V. Samrot^8^, Akon Higuchi^9,10^ and S. Suresh Kumar ^1,11*^” it should be “Mahendran Maniam^8^, Antony V. Samrot^9^, Akon Higuchi^10,11^ and S. Suresh Kumar^1,12*^.”

The authors apologize for this error and state that this does not change the scientific conclusions of the article in any way. The original article has been updated.

