# Supplementary material for: Stem Cell Therapy in Dengue Virus-Infected BALB/C Mice Improves Hepatic Injury
Source: Front Cell Dev Biol. 2021 Jul 5;9:637270. doi: 10.3389/fcell.2021.637270 (PMC8287336; doi:10.3389/fcell.2021.637270)
Supplement: Supplementary file 1 [file Table_1.docx]

**Table 1**

| Test id | Gene id | Gene | Locus | Control (FPKM value) | G1 (FPKM Yalue) | Log2(fold change) | q value | Significant |
| --- | --- | --- | --- | --- | --- | --- | --- | --- |
| XLOC_013318 | XLOC_013318 | Rab26os | 17:23031320-23077642 | 0 | 10.1387 | inf | 1 | no |
| XLOC_015110 | XLOC_015110 | Gm10269 | 18:17819189-17819561 | 0 | 16.6601 | inf | 1 | no |
| XLOC_015371 | XLOC_015371 | Gm23119 | 18:72334875-723350 15 | 0 | 150.436 | inf | 1 | no |
| XLOC_016654 | XLOC_016654 | MGP_BALBcJ_G0025497 | 2:25678689-25679096 | 0 | 10.4326 | inf | 1 | no |
| XLOC_0l6928 | XLOC_0l6928 | Gca, MGP_BALBcJ_G0012409, MGP_BALBcJ_G00 | 2:600892 16-60638515 | 0 | 11.9119 | inf | 1 | no |
| XLOC_021941 | XLOC_021941 | 12410 | 3:133780090-134207362 | 0 | 11.5784 | inf | 1 | no |
| XLOC_024848 | XLOC_024848 | 4930431Fl2Rik | 5:42612105-42624487 | 0 | 12.5258 | inf | 1 | no |
| XLOC_025181 | XLOC_025181 | Mthfd21 | 5:89281051-89367605 | 0 | 13.6307 | inf | 1 | no |
| XLOC_029193 | XLOC_029193 | Igkv8-24 | 6:67945643-68111408 | 0 | 16.0968 | inf | 1 | no |
| XLOC_030771 | XLOC_030771 | MGP_BALBcJ_00032442 | 7:83517729-83518781 | 0 | 10.1687 | inf | 1 | no |
| XLOC_031177 | XLOC_031177 | Mylpf | 7:127702053-127707439 | 0 | 120.826 | inf | 1 | no |
| XLOC_031699 | XLOC_031699 | Zfp940 | 7:28205575-2823197 1 | 0 | 27.239 | inf | 1 | no |
| XLOC_033124 | XLOC_033124 | MGP_BALBcJ_00004533 | 8:66603202-66686224 | 0 | 18.0628 | inf | 1 | no |
| XLOC_035606 | XLOC_035606 | Gm44335 | 9:41376750-41378134 | 0 | 574.563 | inf | 1 | no |
| XLOC_036555 | XLOC_036555 | MGP_BALBcJ_00014446 | X:6 l 059408-61059666 | 0 | 23.7736 | inf | 1 | no |
