# Supplementary material for: Stem Cell Therapy in Dengue Virus-Infected BALB/C Mice Improves Hepatic Injury
Source: Front Cell Dev Biol. 2021 Jul 5;9:637270. doi: 10.3389/fcell.2021.637270 (PMC8287336; doi:10.3389/fcell.2021.637270)
Supplement: Supplementary file 2 [file Table_2.docx]

**Table 2**

| **Test ID** | **Gene ID** | **Gene** | **Locus** | **Control (FPKM value)** | **G2 (FPKM value)** | **Log2(fold change)** | **q value** | **Significant** |
| --- | --- | --- | --- | --- | --- | --- | --- | --- |
| XLOC_003186 | XLOC_003186 | Sult3a 1 | 10:31283423-3 1298517 | 0 | 48.8338 | inf | 0.007606 | yes |
| XLOC_021585 | XLOC_021585 | Hao2 | 3:96548755-96566667 | 0 | 36.539 | inf | 0.007606 | yes |
| XLOC_021924 | XLOC_021924 | MGP_BALBcJ_00006282 | 3:131637308-13 1643455 | 0 | 41.0333 | inf | 0.007606 | yes |
| XLOC_30257 | XLOC_30257 | MGP_BALBcJ_G0040533 | 7:24101650-24108542 | 0 | 52.1268 | inf | 0.007606 | yes |
| XLOC_30258 | XLOC_30258 | Cyp2b9 | 7:24185787-24223302 | 0 | 167.403 | inf | 0.007606 | yes |
| XLOC_030259 | XLOC_030259 | MGP_BALB cJ_G0031867 | 7:24292602-2433421 1 | 0 | 209.279 | inf | 0.007606 | yes |
| XLOC_031499 | XLOC_031499 | Sult2a3 | 7:11494777 -11551540 | 0 | 34.5995 | inf | 0.007606 | Yes |
| XLOC_000447 | XLOC_000447 | Gm27512 | 1:61169239-61202084 | 0 | 436.136 | inf | 1 | no |
| XLOC_000503 | XLOC_000503 | Gm25360 | 1:70252534-70252725 | 0 | 94.1518 | inf | 1 | no |
| XLOC_001981 | XLOC_001981 | MGP_BALBcJ_G0010132 | 1:58438442-58523586 | 0 | 33.326 1 | inf | 1 | no |
| XLOC_002729 | XLOC_002729 | Fmo3 | l :161974097-162100359 | 0 | 110.528 | inf | 0.338451 | no |
| XLOC_003112 | XLOC_003112 | MGP_BALBcJ_G0036352 | 10:15144200-15155939 | 0 | 29.8837 | inf | 1 | no |
| XLOC_003209 | XLOC_003209 | Rnu3a | l 0:37833676-37833890 | 0 | 116.346 | inf | 1 | no |
| XLOC_005084 | XLOC_005084 | Gml7305 | 11:67460712-67487429 | 0 | 10.2884 | inf | 1 | no |
| XLOC_005422 | XLOC_005422 | MGP_BALBcJ_G0018893 | 11:92952527-92956552 | 0 | 10.5147 | inf | 1 | no |
| XLOC_007533 | XLOC_007533 | Acot3 | 12:81841321-81863070 | 0 | 30.5984 | inf | 0.478207 | no |
| XLOC_007547 | XLOC_007547 | Gm 17139 | 12:82747202-82820080 | 0 | 14.7918 | inf | 1 | no |
| XLOC_012625 | XLOC_012625 | Gml 0076 | 16:468038-468405 | 0 | 14.5565 | inf | 1 | no |
| XLOC_013281 | XLOC_013281 | Gml0226 | 17:19869811-19870066 | 0 | 10.0665 | inf | 1 | no |
| XLOC_014038 | XLOC_014038 | Snora78 | 17:23277562-2327 7996 | 0 | 377.441 | inf | 1 | no |
| XLOC_014192 | XLOC_014192 | MGP_BALBcJ_G0003360 | 17:32930035-353 87947 | 0 | 17.1602 | inf | l | no |
| XLOC_016013 | XLOC_016013 | Slc22a26 | 19:4653942-4675569 | 0 | 19.7565 | inf | l | no |
| XLOC_016549 | XLOC_016549 | mt-Tsl | 2:19329205-19618562 | 0 | 288021 | inf | l | no |
| XLOC_017204 | XLOC_017204 | MGP_BALBcJ_G00364 14 | 2:88663442-88687150 | 0 | 11.3819 | inf | l | no |
| XLOC 017433 | XLOC 017433 | MGP_BALBcJ_G0011813 | 2:119439014-119486495 | 0 | 10.9909 | inf | 1 | no |
| XLOC_018260 | XLOC_018260 | MGP_BALBcJ_G0036413 | 2:26820995-26941552 | 0 | 19.9283 | inf | l | no |
| XLOC_020938 | XLOC_020938 | Gm33051 | 3:25629719-25640724 | 0 | 37.8406 | inf | 1 | no |
| XLOC_022066 | XLOC_022066 | Gm24494 | 3:151934096-151939819 | 0 | 52395.6 | inf | 1 | no |
| XLOC_023192 | XLOC_023192 | Gm l3203 | 4: 146653969-146656880 | 0 | 10.6962 | inf | 1 | no |
| XLOC_024169 | XLOC_024169 | Gm26716 | 4: 127942608-127976912 | 0 | 12.0673 | inf | 1 | no |
| XLOC_029178 | XLOC_029178 | Igkv5-39 | 6:67607309-67607863 | 0 | 11.8116 | inf | 1 | no |
| XLOC_029675 | XLOC_029675 | Gm44096 | 6:122520707-122613222 | 0 | 17.595 | inf | l | no |
| XLOC_030266 | XLOC_030266 | Cyp2gl | 7:24971909-24994733 | 0 | 18.6062 | inf | 1 | no |
| XLOC_031497 | XLOC_031497 | MGP_BALBcJ_G003165S,Sult2a2 | 7:11303713-11394048 | 0 | 177.379 | inf | 0.338451 | no |
| XLOC_031503 | XLOC_031503 | Sult2a6 | 7: 11656322-11692614 | 0 | 153.433 | inf | 0.338451 | no |
| XLOC_033861 | XLOC_033861 | Gm28063 | 8:54953411-54956948 | 0 | 14.4561 | inf | 1 | no |
| XLOC_034394 | XLOC_034394 | Gm24357 | 9: 12002860-12002934 | 0 | 145893 | inf | 1 | no |
| XLOC_035890 | XLOC_035890 | Rn7sk | 9:75583363-75583694 | 0 | 11.4497 | inf | 1 | no |
